# The Role of Artificial Intelligence in Providing Real-Time Guidance During Interventional Cardiology Procedures: A Narrative Review

**DOI:** 10.7759/cureus.83464

**Published:** 2025-05-04

**Authors:** Venkata K Yannakula, Amruth A Alluri, Dany Samuel, Simisolaoluwa A Popoola, Bashir A Barake, Alwaleed Alabbasi, Abdishakur S Ahmed, David A Cortes Bandy, Nusrat J Jesi

**Affiliations:** 1 Medical School, Kasturba Medical College, Manipal, Manipal, IND; 2 Internal Medicine, American University of the Caribbean School of Medicine, Cupecoy, SXM; 3 Radiology, Medical University of Varna, Varna, BGR; 4 School of Medicine, Duke University School of Medicine, Durham, USA; 5 Gilbert and Rose-Marie Chagoury School of Medicine, Lebanese American University School of Medicine, Byblos, LBN; 6 Medical School, Royal College of Surgeons in Ireland, Dublin, IRL; 7 Medicine and Surgery, Tanta University, Tanta, EGY; 8 Internal Medicine, Universidad Europea de Madrid, Madrid, ESP; 9 Internal Medicine, Shaheed Syed Nazrul Islam Medical College, Kishoreganj, BGD

**Keywords:** angioplasty and stenting, artificial intelligence in cardiology, cardiology research, coronary artery bypass grafting(cabg), interventional cardiology, intravenous ultrasound (ivus), pacemaker placement, tavr( transcatheter aortic valve replacement)

## Abstract

Integrating artificial intelligence (AI) in interventional cardiology revolutionizes procedural guidance, particularly in high-stakes environments such as angioplasty and stent placement. In this narrative review we explore the role of AI in providing real-time decision support, enhancing precision, and improving patient outcomes during these complex procedures. AI algorithms can identify critical anatomical features, predict complications, and optimize stent positioning with unprecedented accuracy by analyzing data from imaging modalities like intravascular ultrasound and optical coherence tomography. The findings of this narrative review, from which we have reviewed more than 150 studies across multiple databases, highlight the necessity of continued research and development to utilize AI to its full potential in enhancing the efficacy and safety of interventional procedures. In this review we highlight AI’s current advancements, challenges, and potential in real-time interventional cardiology procedures, emphasizing its transformative impact on clinical practice and patient care.

## Introduction and background

**Figure 1:**
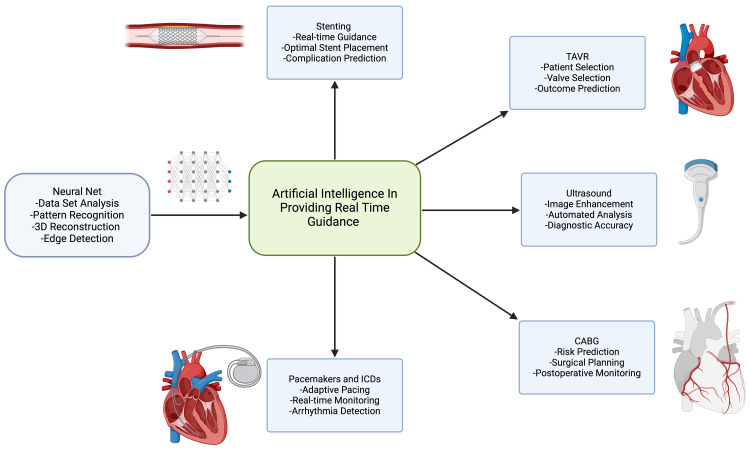
Scope of AI in patient management TAVR: transcatheter aortic valve replacement, CABG: coronary artery bypass graft, ICD: implantable cardioverter-defibrillator Image credits: Amruth A. Alluri

Artificial intelligence (AI) is an umbrella term that refers to the broad application of analytical algorithms to provide machines with the capacity for reasoning and to perform cognitive tasks such as word and object recognition, problem-solving, decision-making, and using language by learning from the data iteratively and enabling computers to uncover hidden insights without explicit programming as shown in Figure 1. AI, including deep learning (DL) and robotics, integrates and analyzes complex biomedical data [1,2].

**Figure 2:**
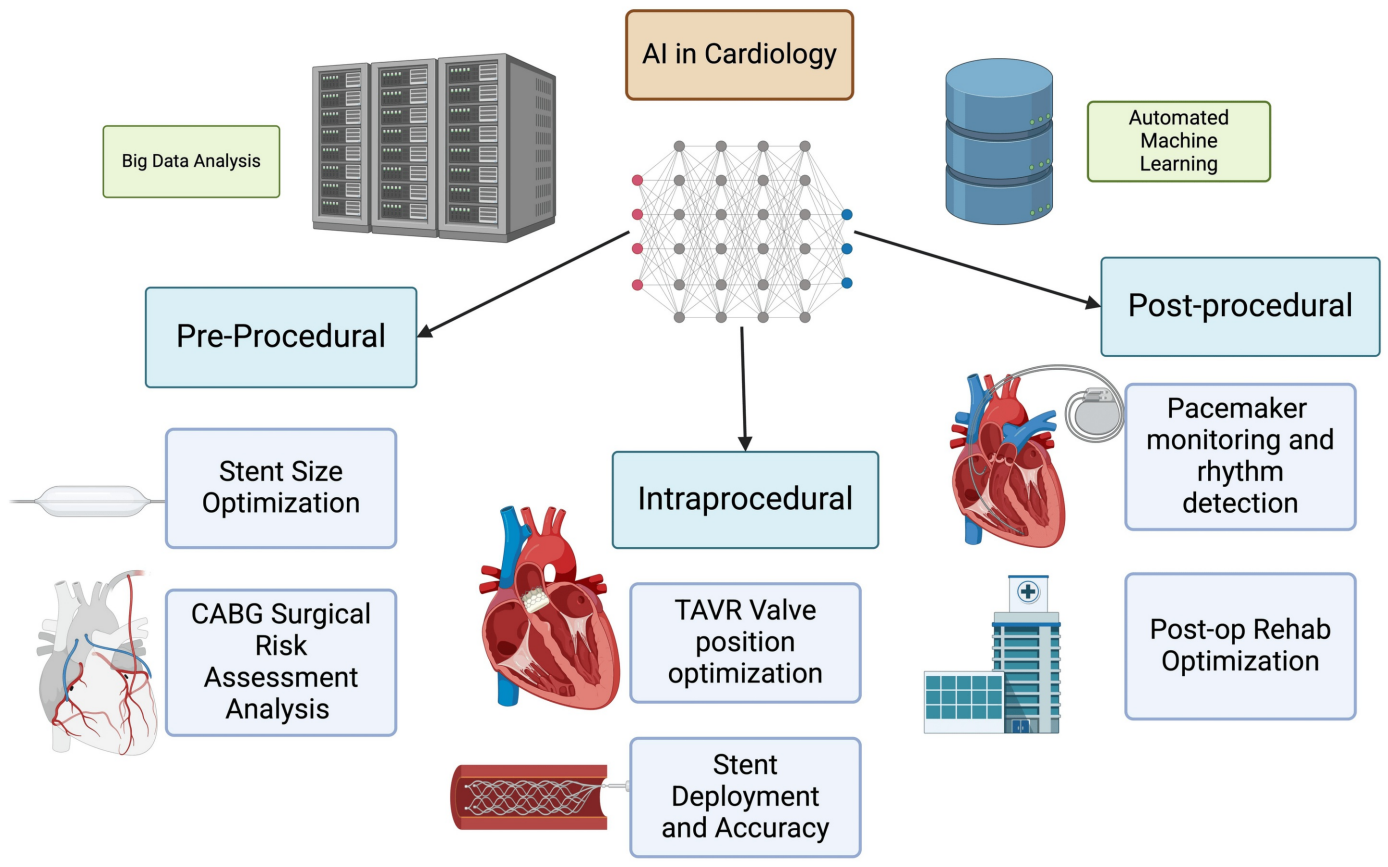
Applications of AI in cardiology during pre-, intra-, and post-procedure TAVR: transcatheter aortic valve replacement, CABG: coronary artery bypass graft Image Credits: Amruth A. Alluri

AI’s role in medicine remains in its early stages, lagging in fields such as financial technology and aerospace. AI technologies could enhance real-time clinical decision-making through better pattern recognition, improve workflow in catheterization labs, standardize catheter procedures with robotics, and offer personalized risk assessments and guidance throughout interventions such as angiography and stent placement [2-4]. Even though AI and DL are extremely capable of identifying patterns from data, they are not exempt from interpretation errors, which can result in an inaccurate classification of information. Some changes may be so subtle that they could escape human perception, and this can perplex the best-operating machine learning (ML) algorithms and can have detrimental effects on health care. Additionally, issues such as limited generalizability, ethical concerns, and data privacy pose obstacles to clinical implementation [4,5]. In this review we will address the applications of AI algorithms across three procedural stages as shown in Figure 2.

## Review

Angioplasty

Pre-procedural Applications

Enhancing diagnosis of coronary artery disease with AI: AI is significantly enhancing the assessment and planning of angioplasty by improving the diagnosis of coronary artery disease (CAD). AI-based systems create 3D reconstructions of coronary arteries from angiographic images, allowing for better evaluation of stenoses and their hemodynamic significance through virtual fractional flow reserve (FFR). AI can help decide when specialised tools like balloons or atherectomy devices are needed, and new AI models can estimate FFR non-invasively, though these still need thorough testing and regulatory approval before they become standard practice. Some promising results, like the 94% accuracy reported by Min et al., are based on internal tests and need to be confirmed in larger, more diverse patient groups. In real-world settings, differences in imaging equipment and operator technique can affect how well these models work. Clinicians may also find it challenging to trust automated measurements unless the AI provides clear, interpretable outputs. Looking ahead, combining data from multiple imaging techniques (like intravascular ultrasound (IVUS) and optical coherence tomography (OCT)) could boost accuracy even further. While AI is already good at spotting general plaque buildup, detecting high-risk, “vulnerable” plaques remains a tougher and more important challenge. Finally, many current studies rely on small, single-center datasets, which limits how broadly their findings can be applied. Tools like HeartFlow Fractional Flow Reserve - Computed Tomography (FFRCT; HeartFlow, Inc., Mountain View, CA, USA) provide highly accurate assessments, achieving over 90% sensitivity and specificity compared to traditional methods. IBM’s Medical Sieve (San Jose, CA, USA) automatically identifies coronary stenoses in angiograms, aiding treatment decisions for angioplasty [6]. Coronary artery calcification (CAC) refers to calcific lesions in the coronary arteries, which can be asymptomatic or lead to flow-limiting stenosis, resulting in calcified CAD. Calcified lesions are generally more stable and associated with a lower risk of major adverse cardiovascular events (MACE) compared to noncalcified lesions. However, percutaneous coronary interventions (PCIs) for calcified lesions pose unique challenges, including higher MACE rates due to stent under-expansion and increased technical complexity. CAC scores have traditionally been determined through cardiac computed tomography (CT), relying on manual input to detect calcified lesions. A new AI-based model for fully automated CAC scoring has been developed and tested on an independent dataset of CT scans, demonstrating strong correlation and agreement with manual assessments. This AI approach can enhance the identification and quantification of CAC, significantly decreasing the time needed for human analysis [7].

These applications illustrate how AI can significantly improve the safety, efficiency, and effectiveness of angioplasty and PCI procedures. Reliable assessment of CAD is vital for deciding on invasive procedures like PCI or coronary artery bypass graft (CABG) surgery [8]. Traditional methods, relying on visual interpretation of coronary angiography (CAG), lack standardization and exhibit significant observer variability [9]. The DeepCoro algorithmic pipeline was developed to automatically evaluate stenosis severity in CAG videos, utilizing temporal data for improved accuracy. It features advanced algorithms for anatomical detection, stenosis localization, and severity prediction, aiming to match or exceed cardiologists’ diagnostic accuracy. By benchmarking against existing methods like CathAI, DeepCoro enhances efficiency and accuracy in CAD assessments, potentially transforming clinical practice in evaluating coronary stenoses [10].

Tailoring interventions through machine learning: ML techniques also predict plaque vulnerability by analyzing diverse imaging data, which helps tailor interventions. Recent software, such as Abbott’s Ultreon 1.0 (Abbott Park, IL, USA), utilizes DL to analyze OCT images in real time, quantifying calcium burden to optimize stent sizing and positioning [6]. This factor assesses the physiological impact of coronary artery stenosis and how it affects the blood flow to the myocardium and are obtained by measuring the blood pressure before and after stenosis while inducing the highest amount of perfusion possible by using drugs such as adenosine. CAC scoring informs prognosis after stenosis detection. It’s important to distinguish between natural progression risk and the risks introduced by intervention. To interpret FFR values, note that FFR ≤ 0.80. This suggests that the stenosis is significant, meaning the reduction in perfusion is severe enough to require methods for revascularization of myocardium such as angioplasty while FFR > 0.80. This indicates that the stenosis is not significantly impacting perfusion, and interventional cardiology methods are not currently required. In a study, Ariel Rougin et al. used AutocathFFR, which was an ML algorithm taught to measure FFR based on data from a training group and correlate them to angiographies given to the model now [11]. The data given to the algorithm were highly refined to remove low-quality images and damaged data while also keeping the data fed to the algorithm that were within the parameters to which the algorithm had already been exposed. This was to limit systematic errors because the study would have been uncontrolled if the algorithm had been suddenly exposed to a wider criteria of data, as well as to deliver an accurate diagnosis by predicting the FFR value [12].

Given the study was also conducted with a very small sample size of 31 subjects, authors of further studies should greatly increase the sample size and allow for more errors for the algorithm to reduce errors. The accuracy of the AutocathFFR algorithm compared to traditional wire-based FFR is similar, allowing scope for further studies to increase its efficacy and applications [11]. Supervised learning trains algorithms to predict patient responses to treatments, whereas unsupervised learning identifies patterns in unlabeled data. Clustering techniques, a form of unsupervised machine learning, group patients into subgroups based on shared clinical, imaging, or genetic characteristics, help us identify more homogeneous patient populations leading to the exposure of several distinct risk profiles or treatment responses and thus enabling clinicians to tailor therapies more precisely for every subgroup population [8]. A recent study demonstrates that utilizing an AI-enhanced convolutional neural network (CNN) model to analyze standard 12-lead ECGs allows ECG to function as an effective screening tool for detecting significant CAD and pinpointing the location of coronary blockages. This approach can be easily integrated into health screenings for asymptomatic patients, helping to identify those at higher risk for future coronary events [13].

Risk prediction and management in angioplasty:AI has applications in risk prediction for patients undergoing angioplasty. By integrating diverse health factors-such as comorbidities, socioeconomic status, and angiographic details-machine learning models can accurately forecast complications and identify patients at higher risk for adverse events, including bleeding, acute kidney injury, and mortality. However, the impact of each influencing factor varies: for example, dataset heterogeneity can strongly undermine the generalisability of AI predictions, potentially leading to less reliable risk assessments in diverse patient populations. Operator and equipment variability moderately affect the consistency of AI-driven measurements, which may influence real-time decision-making during procedures. Meanwhile, lack of model explainability and limited external validation can significantly restrict clinical adoption, as clinicians may be less likely to trust or act on AI-generated recommendations without clear interpretability or proven accuracy across settings. Addressing these limitations is essential to fully realise the benefits of AI in optimising outcomes for angioplasty patients [14]. Overall, AI applications are streamlining diagnostic and pre-procedural planning processes in angioplasty, leading to improved patient care and outcomes.

Intra-procedural Applications

In the first study being reviewed, Yaya Sakakura et al. used a real-time artificial assistance software during carotid artery stenting wherein it guides the tracking wires, catheters, and other devices and the software automatically provides a notification when the device moves out of place. The assistance software used in this study is called Neuro-Vascular Assist, along with an embolism protection system called FilterWire EZ. The Neuro-Vascular Assist system integrates into the angioplasty workflow as a passive monitoring tool: it continuously tracks the position of guidewires and catheters in the background and only alerts the operator if a device deviates from its intended path, leaving all adjustments to the clinician. This approach supports operator autonomy and minimises workflow disruption, but does not provide active, real-time device guidance. While early results were promising, with precision of 0.82 and recall of 0.94, these findings are based on just six patients of an average age of 80 years. Only six cases were taken, which is too less, but the early indication of the high efficacy in the algorithm means that further studies can be done, and given this program receives angiography input as well, we can refine it using other asserted algorithms. More large-scale studies are primarily required to uphold the credibility and reliability of the software [15,16].

Post-procedural Applications

An AI-based algorithm is being developed for comprehensive three-dimensional (3D) reconstruction of the coronary artery tree from multiple angiographic images. This process involves detecting vessel bifurcations as key landmarks for correlating data across views and using optimal matching to create a complete arterial model. Additionally, a real-time FFR computation algorithm will utilize pre-PCI anatomical and stent information to provide virtual post-PCI FFR values, enhancing efficiency compared to prior methods that required virtual stenting, with validation through invasively measured post-PCI FFR data [10]. By providing immediate functional assessment of coronary lesions, real-time FFR helps clinicians make faster, more informed decisions, which can reduce unnecessary interventions and shorten overall procedure times, ultimately leading to better patient outcomes. We reviewed a study conducted by Kim A. Eagle et al., who used these artificial neural networks to predict in-hospital death after percutaneous transluminal coronary angioplasty (PCTA), which is a type of noninvasive coronary angioplasty. It should be noted that the neural networks used in the Kim A. Eagle et al. study were developed in the 1990s, before the advent of modern deep learning techniques and with far less computational power, so their performance and interpretability may not match current AI models. In this study, the authors analysed data from 3,019 angioplasty procedures performed between 1994 and 1997. They collected 38 clinical variables, including patient history, presentation, and procedural details, which served as input features for the development of 60 artificial neural network models. These models were constructed in an unguided (unsupervised) fashion to predict in-hospital outcomes based on the available risk factors.. They divided the 3,019 procedures into a training group, which helped them input the data into the artificial neural networks so that they could learn from some data and use compiled data to predict in-hospital death after PTCA for the remaining 1,465 procedures, which were put together as a validation group [17].

The last application we will be looking at is the use of ML, particularly in the prediction of in-hospital bleeding after 72 hours post procedure. This kind of major bleeding being investigated in this study is a serious complication after the completion of percutaneous coronary angioplasty, and the major application of being able to predict such bleeds is to reduce postoperative mortality and take preventive action accordingly. We collected data from 2009-2015, including a total of 3,316,465 PCI procedures. For 149,724 cases with postoperative bleeds, we classified them as major post-PCI bleeds and used concordance statistics to rank the cases based on predictive score of postoperative PCI bleeding. We also compared each patient’s case to check if the prediction values from C statistics correctly predicted the postoperative bleed [11]. Table 1 shows the various patient comorbidities from the study.

**Table 1:** Patient characteristics and medical conditions considered as risk factors Taken from JAMA open access [11] PCI: percutaneous coronary intervention, CABG: coronary artery bypass graft

Characteristic	Full Sample (3,316,465)	Bleeding Cases (149,724)	Nonbleeding Cases (3,166,741)
Post-PCI major bleeding rate, %	4.51	100	0
Age, median (IQR)	65 (56-73)	68 (59-77)	65 (56-73)
Male sex, %	68.1	50.6	68.9
BMI, median (IQR)	29.1 (25.7-33.3)	27.7 (24.3-32.1)	29.2 (25.8-33.4)
Medical conditions			
Diabetes, %	37.0	37.5	36.9
Hypertension, %	82.1	80.2	82.2
Peripheral vascular disease, %	12.2	15.2	12.0
Chronic kidney disease, %	3.7	9.9	3.5
Previous PCI, %	41.2	30.0	41.7
Previous CABG, %	18.1	14.6	18.3

The risk score, which was already being used, gave a value of mean C statistic of 0.77, with a range of 0.77-0.77, and the most notable change occurred when the XGBoost algorithm was used to increase the mean concordance statistic to 0.81 (range: 0.80-0.81). Out of the 14,729 bleeding cases, 105,316 were predicted correctly. A higher mean C statistic reflects better discrimination between patients who will and will not experience an adverse event, so the increase with XGBoost indicates improved predictive accuracy for clinical outcomes. The prediction figures are shown in Table 2 below [11].

**Table 2:** A statistical comparison of the outcomes from the existing simplified risk score and blended model Taken from JAMA open access [11]

Model used		True-Positives	True-Negatives	False-Positives	False-Negatives	Mean standard deviation threshold
Existing simplified risk score	Existing simplified risk score with high-risk threshold	105 316 (3.1%)	2 208 569 (66.5%)	958 172 (28.9%)	44 408 (1.3%)	65.0 points (6.5%)
	Existing simplified risk score with data-driven threshold*	47 445 (1.4%)	2 990 509 (90.1%)	176 232 (5.3%)	102 279 (3.0%)	96.4 points (14.9%-17.0%)
Blended model	Existing full model	49 967 (1.5%)	2 982 389 (89.9%)	184 352 (5.5%)	99 757 (3.0%)	11.9% (0.5%)
	Blended model with lasso regularisation	51 840 (1.5%)	2 977 168 (89.7%)	189 573 (5.7%)	97 884 (2.9%)	11.6% (0.4%)
	Blended model with gradient descent boosting*	55 527 (1.6%)	3 013 868 (90.8%)	152 873 (4.5%)	94 197 (2.8%)	15.6% (0.9%)

We limit the discussion by comparing the two sets on the second column in asterisks. The XGBoost blended model had predicted fewer true positives (1.6%) than the existing simplified risk score model (3.1%), but the number of true negatives was much more than the existing model (90.8% vs 66.5%). Both the false and true negatives were also significantly lower than the existing risk model, meaning that the newer blended XGBoost model has much more accuracy, which is shown by the higher standard deviation of 15.6% compared to 6.5%, more than double, indicating the increased accuracy. In this study we only investigated bleeding post procedure, but using data from primarily the degree of stenosis such as a coronary calcification score could help in the prognosis after angioplasty and assessment risk of postoperative bleeding and can expand the to introduce more training datasets to the models used to gain more exposure and to increase accuracy of already developed models. These advances in prediction can help reduce reliance on invasive monitoring and enable earlier, targeted interventions for high-risk patients, translating to more efficient and personalized post-angioplasty care [17].

Stenting in coronary angioplasty

AI enhances stent placement in coronary angioplasty by providing real-time guidance for precise implantation using angiographic images, leading to improved accuracy, reduced restenosis risk, and better patient outcomes. AI plays a crucial role in guiding interventional cardiologists during stent deployment in coronary angioplasty, a minimally invasive procedure for treating clogged coronary arteries. Manually placing a stent can be complex and may lead to complications such as embolisation or perforation. To avoid overgeneralisation, it is important to distinguish between general applications of AI-such as automated data analysis and decision support-and specific types of AI tools. For example, supervised ML models are commonly used for structured data and outcome prediction, while DL imaging models, including convolutional neural networks, excel at interpreting complex imaging data like intravascular ultrasound or OCT. Although the primary focus here is on stenting, CABG risk prediction models are included for comparison because many patients with complex coronary artery disease may be candidates for either procedure. Understanding how AI enhances risk stratification in both settings helps inform optimal treatment selection and highlights the broader impact of AI across interventional cardiology.

Furnary et al. (2020) found that AI-assisted stent implantation results in better stent positioning and a lower risk of MACE compared to manual methods. Another study indicated that AI could reduce restenosis rates by up to 30% [18]. As AI continues to be integrated into catheterization labs, it enhances the precision of stent placement, improving patient outcomes and minimizing adverse events. Interventional cardiologists increasingly rely on AI for more accurate and effective coronary angioplasty procedures. Table 3 below gives an overview regarding the various components of stenting in Coronary angioplasty and each of their own AI advanced techniques along with each of the various benefits.

**Table 3:** On overview highlighting the various applications of stenting compared to traditional methods CABG: coronary artery bypass graft, IVUS: ⁠intravascular ultrasound guided procedure, OCT: optical coherence tomography, AUC: area under the curve

Aspect	Traditional Method	AI-Enhanced Method	Improvement Metrics
Stent Placement Precision [19]	Manual positioning based on angiography	Al-guided optimization using imaging data	Reduced restenosis rates by up to 30%
Procedure Time [20,21]	Longer due to manual assessments	Shortened with real-time Al analysis	Procedure time decreased by 15-20%
Risk Prediction in CABG [22]	Standard risk scores (e.g. EurosCORE )	Al-based predictive models using big data	Improved accuracy (AUC increased from 0.76 to 0.85)
Imaging Interpretation (IVUS/OCT) [23,24]	Manual interpretation by specialists	Automated analysis via Al algorithms	Reduced interpretation time by 50%, increased diagnostic accuracy
Pacemaker Programming [25]	Fixed parameter settings	Adaptive algorithms for personalized settings	Enhanced patient outcomes, reduced hospital readmissions

Pacemaker placement ⁠

Cardiac pacemakers are devices that regulate heartbeats in patients with arrhythmias. Their use has surged globally, with about 1,000,000 implants annually, driven by an aging population [26]. AI-powered pacemakers can analyze real-time data, adjusting pacing parameters based on individual patient needs, enhancing efficacy and outcomes [27]. Traditional pacemakers operate with static, pre-set pacing parameters, while AI-powered pacemakers use adaptive algorithms to automatically adjust pacing in real time based on the patient’s physiological needs, offering a more personalized approach to rhythm management. AI algorithms can monitor heart rhythms and vital signs, continuously enabling timely interventions [28]. A DL model has shown high accuracy in predicting future pacemaker implantation and related cardiovascular events using ECG data [28]. Additionally, AI can optimize battery life by adjusting power consumption based on activity levels, thus extending device longevity [29]. By optimizing pacing and power consumption, AI-enabled devices can extend battery life, which translates to fewer surgical procedures for device replacement and reduced long-term risk for patients. AI models can analyze standard ECGs to detect subtle early signs of conduction system disease, enabling clinicians to identify patients at risk for future pacemaker implantation before overt symptoms develop. Despite these benefits, challenges such as regulatory requirements, ethical concerns regarding data use, and the need for clinical validation must be addressed. Table 4 below shows the key studies that have investigated the integration of AI into cardiac pacemakers. Regulatory and clinical adoption pathways differ for AI in stenting and pacemakers: stenting AI tools often require device-specific validation and approval for procedural support, while pacemaker AI must meet stringent safety and efficacy standards for implantable devices, with both facing ongoing scrutiny as adaptive algorithms evolve post-approval. In addition, procedural AI for stent placement raises important ethical considerations around clinician trust in automated recommendations and legal liability in the event of adverse outcomes, which must be addressed for safe integration into practice.

**Table 4:** Key Studies on AI Integration with Cardiac Pacemakers AUC: area under the curve, PMI: pacemaker implantation, ICD: implantable cardioverter-defibrillator

Study	Year	AI Technique Used	Sample Size	Outcomes Measured	Key Findings
Hung et al. [21]	2024	Deep Learning Model using ECG Data	10,545 patients	Prediction of PMI and cardiovascular events	High accuracy in predicting PMI within 30, 60, and 90 days with AUC values of 0.870, 0.878, and 0.883, respectively; significant predictive capabilities.
Dey et al. [27]	2024	Advanced Controller Design for Pacemakers	Comprehensive Review	Pacing control improvements	AI enhances pacing control and device functionality; provides adaptive and personalized pacing strategies.
Alugubelli et al. [28]	2022	AI in Wearable Devices for Monitoring	Review of Current Tech	Heart rate and variability analysis	AI-enabled wearables improve remote monitoring and early detection of cardiac anomalies, aiding in timely interventions.
Sahu et al. [30]	2023	Evolution of Pacemakers and ICDs	Literature Review	Device advancements over time	AI contributes to significant advancements in pacemaker size, functionality, and adaptability.

Transcatheter valve replacement

AI is revolutionizing cardiology, particularly in transcatheter aortic valve replacement (TAVR). It enhances patient selection, diagnosis, and treatment, accelerating both diagnostic and therapeutic processes. AI provides real-time imaging and supports improving procedural success rates and reducing complications, making TAVR a more viable alternative to surgical aortic valve replacement due to increased precision and better outcomes over a wider range of patients at risk [31,32]. For context, recent studies show that one-year survival is slightly higher for surgical aortic valve replacement (SAVR) at 64.2%, compared to 63% for TAVR, with differences becoming more pronounced over time. AI applications in TAVR can be broadly categorized as either diagnostic - such as early screening for aortic stenosis using ECG or imaging - and procedural support, including intraoperative guidance and real-time imaging enhancement during the intervention. These are distinct uses, each with unique technical and clinical requirements.

AI is proving valuable in screening for severe aortic stenosis, using electrocardiograms (EKGs) as an initial assessment tool rather than just a diagnostic method as seen in Table 5, shows studies summarized in the span from 2013 to 2023 reflecting the rapid evolution of AI sophistication - from early semi-automated tools to advanced deep learning models now capable of fully automated image analysis and Table 6 below, intraoperative support includes real-time features such as motion tracking, anatomical overlays, and automated device positioning, demonstrating the technical depth of current AI involvement. Additionally, AI can streamline the segmentation of 3D CT images, enhancing the planning and execution of treatments and interventions with greater speed and accuracy [36,37]. While AI can enhance ECG-based screening for aortic stenosis, a positive screen still requires confirmation with standard imaging modalities such as echocardiography or CT, as AI does not yet replace imaging for definitive diagnosis.

**Table 5:** Key Studies of AI applications in TAVR Procedures TAVR: transcatheter aortic valve replacement, AUC: area under the curve

Study	Year	AI Technique Used	Application	Key Findings
Kwon et al. [33]	2020	Deep learning on ECG data	Diagnosis of aortic stenosis	AI algorithm accurately detected aortic stenosis using ECGs, enabling early screening and diagnosis.
Watanabe et al. [34]	2013	Automated 3D CT assessment	Valve sizing and annulus measurement	AI improved the precision of aortic annulus measurements, aiding in appropriate valve selection for TAVR.
Astudillo et al. [35]	2019	Machine learning for device sizing	Automated device selection	Enabled automated device size selection, reducing sizing errors and improving patient outcomes.
Lou et al. [36]	2015	Semi-automated and fully automated measurements	Pre-procedural planning	Demonstrated interchangeability between manual and AI-assisted measurements, supporting the reliability of AI tools in clinical practice.
Judson et al. [20]	2023	Supervised random forest machine learning	Predicting length of hospital stay	AI model outperformed traditional models (AUC 0.82 vs. 0.65) in predicting short and extended hospital stays post-TAVR, identifying new predictive factors.

**Table 6:** Comparison of Traditional vs. AI-Assisted TAVR Processes TAVR: transcatheter aortic valve replacement

Process Stage	Traditional Approach	AI-Assisted Approach	Advantages of AI Integration
Diagnosis [33]	Reliant on symptoms, physical exams, and echocardiograms [33]	Uses AI to analyze ECGs and imaging for early detection [33]	Earlier diagnosis, improved accuracy [33]
Patient Selection	Based on standard risk assessments and guidelines [38,39]	AI evaluates multiple factors to identify suitable candidates [38,39]	Expanded eligibility, personalized risk assessment [38,39]
Pre-Procedural Planning [36,37]	Manual measurements from CT scans for valve sizing [36,37]	AI automates image segmentation and measurements [36,37]	Increased precision, reduced human error [36,37]
Outcome Prediction [20]	Utilizes traditional statistical models [20]	AI predicts complications and hospital stay durations [20]	Proactive management, resource optimization [20]
Intraoperative Support [40]	Dependent on operator experience and standard imaging [40]	AI provides real-time decision support and imaging enhancement [40]	Enhanced safety, reduced procedure times [40]

Pre-procedural Planning

To ensure patient safety, CT is now recommended as a crucial initial investigation for TAVR patients. It provides detailed anatomical information for valve selection and sizing, while also assessing potential complications like annular rupture. These algorithms reduce human error and enhance patient-specific treatment strategies by precisely measuring the aortic annulus and aiding in device sizing through advanced ML techniques [34,36]. Despite improvements in hospital stay duration, disparities remain in achieving early discharge goals. Madison et al. developed a supervised random forest ML model using data from 9,360 outpatient TAVR procedures to identify factors associated with short (<36 hours) and extended (≥72 hours) stays. Their ML model demonstrated greater predictive accuracy (area under the curve (AUC) 0.82 and 0.85) compared to traditional multivariate models (AUC 0.65). In the Madison et al. model, ‘new predictors’ - such as procedure duration and need for post-procedure care - can inform changes to discharge protocols and resource allocation, directly impacting clinical management.” Notably, it identified new predictors, including surgery duration and the need for post-procedure care [41]. One key concern is publication bias in AI research, where positive outcomes are more frequently published, potentially leading to an overestimation of the technology's effectiveness and applicability [40]. Publication bias, where positive results are more likely to be published, may inflate perceived AI effectiveness and contribute to hesitancy or delays in real-world deployment and CT imaging carries limitations, such as the risk of contrast-induced nephropathy in patients with kidney disease. Emerging AI applications are also being explored for MRI and fusion imaging, which may further enhance pre-procedural planning in TAVR for example, AI can facilitate the fusion of fluoroscopy and CT datasets to provide enhanced anatomical visualization during valve deployment. Beyond technical improvements, AI integration has also been associated with better patient-centered outcomes, such as improved quality of life and reduced rehospitalization rates following TAVR.

Coronary artery bypass graft surgery

CABG is a major surgical procedure involving the bypass of atheromatous lesions with harvested venous or arterial conduits to restore blood flow to the ischemic myocardium [41]. Traditional risk assessment methods are often criticized for being subjective and may overlook patient-specific nuances. The ability of AI to analyze vast amounts of data can enhance the precision of risk categorization, decision-making, and surgical planning and ultimately improve patient outcomes. In the meta-analysis by Benedetto et al., machine learning models achieved a pooled C-statistic of 0.88 (95% CI: 0.83-0.93), compared to 0.81 (95% CI: 0.77-0.85) for logistic regression, indicating better discrimination for mortality prediction after cardiac surgery. The authors calculated the discriminative performance of both models through the concordance statistics (C-statistics), which scored higher numbers in the ML model compared to the LR model. Those results indeed showed better performance and more accurate mortality predictions by the ML models [22]. In addition to risk prediction, emerging AI applications in CABG include operative decision-making support, automated graft selection based on patient-specific anatomy, and intraoperative navigation using real-time imaging and computer vision to enhance surgical precision and teamwork.

Tang et al. tested four models for assessment of mortality rates in patients undergoing CABG. The researchers incorporated various types of patients’ data into their AI model including demographic data, clinical history, and laboratory results. The best-performing model overestimated the mortality rates in patients with intermediate risk [42]. While the model overestimated mortality in intermediate-risk patients, this may be an acceptable trade-off if it prompts closer monitoring and preventive care, but it also highlights the need for further calibration and validation in diverse cohorts. In another study, Gaizo et al. evaluated the potential of natural language processing of surgical notes to predict mortality and morbidity. The researchers analyzed the preoperative notes of 1,738 patients from a single institution with three numerical embedding techniques, which were found to surpass the Society of Thoracic Surgeons model, one of the standard models used for predicting risk of complications following cardiac surgery [43]. The study by Gaizo et al. tested several numerical embedding techniques, including basic embedding, ScaleNum, and AttnToNum, with the latter two leveraging advanced context-aware transformations to improve predictive performance in NLP models analyzing surgical notes. Other studies by Khalaji et al. and Gao et al. also revealed that ML methods have satisfactory performances in risk assessment [44,45]. Complex patterns and relationship analysis that can be found in large patient datasets cannot be easily discerned by human analytical experts, whereas AI models can reveal these patterns, translating into better treatments and risk assessment and more effective management of patients’ clinical conditions [46,47].

Overall, our review highlights a paucity of literature whose authors study the use of AI in CABG. All the cited studies’ authors have focused on risk and outcome assessment guiding the patient’s management. Current gaps include a lack of prospective clinical validation studies, limited real-world deployment, and few direct comparisons of AI model predictions against human expert assessments. Currently, most standards of risk assessment including the STS model are based on imputing certain patient information into LR models [48]; furthermore, AI models can be adjusted to cater to other types of populations, improving accuracy of risk assessment in these populations. If AI models are not carefully validated across diverse patient populations, there is a risk of amplifying existing biases, which could worsen disparities in surgical outcomes.

⁠Intravascular ultrasound-guided procedure 

IVUS is a key imaging technique in interventional cardiology, aiding in lesion characterisation, plaque burden assessment, stent placement, and complication identification. However, interpreting IVUS images requires significant time and expertise. AI offers solutions through algorithms that facilitate image processing and automated quantification. By analysing IVUS images before intervention, AI can help tailor lesion preparation strategies, such as identifying when specialty balloons or rotational atherectomy (rotablation) may be needed, and assist in optimal device selection for complex lesions. Table 7 gives us an overview of the various studies that have had successful implementation of AI into IVUS procedures along with their findings, while Table 8 highlights various aspects of IVUS and their AI-enhanced counterparts along with their key findings. 

**Table 7:** Key Studies on AI in IVUS-Guided Procedures FFR: fractional flow reserve, IVUS: intravascular ultrasound

Study	Year	AI Technique Used	Application	Key Findings
Min et al. [19]	2021	Deep learning algorithms	Prediction of post-procedural stent area	- High correlation between predicted and measured stent areas (r = 0.802, p < 0.001)
Lee et al. [49]	2020	Machine learning algorithms	Predicting functional significance (FFR )	- Trained on 1,063 patients - Achieved average diagnostic accuracy of 82% - Combined IVUS features with clinical data
Zhu et al. [24]	2022	Deep learning-based method	Automated lumen and media-adventitia extraction.	- Effective automated segmentation - Reduced need for manual interpretation
Bajaj et al. [50]	2021	Deep learning methodology	Automated detection of end-diastolic frames	- Improved efficiency in identifying frames - Aided in accurate measurements

**Table 8:** Comparison of Traditional vs. AI-Assisted IVUS Analysis FFR: fractional flow reserve, IVUS: intravascular ultrasound

Aspect	Traditional Approach	AI-Assisted Approach	Advantages of AI Integration
Image Interpretation [50]	Requires significant time and expert analysis [50]	Automated quantitation and feature extraction [50]	- Reduces time and expertise needed - Minimizes human error [50]
Stent Area Prediction [51]	Based on operator experience and standard measurements [51]	AI predicts post-procedural stent area [51]	- Helps in pre-planning - Reduces stent failure rates [51]
Functional Significance Prediction [41]	Relies on invasive FFR measurements [41]	AI predicts FFR from IVUS images [41]	- Non-invasive - Reduces procedure time and risks [41]
Detection of Plaques [23]	Manual detection of vulnerable plaques [23]	AI enhances detection using algorithms [23]	- Improves diagnosis - Aids in risk stratification [23]
Frame Selection [43]	Manual identification of end-diastolic frames [43]	AI automates frame detection [43]	- Streamlines workflow - Improves measurement accuracy [43]

Pre-procedure

In another study, June-Goo Lee et al. developed six ML algorithms to predict functionally significant coronary artery blockages (FFR < 0.80), aiding decisions regarding angioplasty or conservative management. In the study, involving 1,063 patients, the authors segmented IVUS images and extracted 99 features, achieving an average diagnostic accuracy of 82% for predicting FFR < 0.80. Though FFR testing is the standard for detecting ischemic lesions, it is costly and carries risks. AI enhances this process by integrating multiple variables to predict ischemia, surpassing traditional statistical methods that struggle with complex interactions. Future researchers could combine mathematical models with AI to improve diagnostic accuracy and clinical decision-making [16,17,50]. In various studies, AI has enhanced the detection of vulnerable plaques, coronary vessel structures, and other critical parameters [18,52]. Since FFR is an established clinical standard, the adoption of non-invasive, AI-derived FFR surrogates will require robust external validation and regulatory approval before they can be routinely used in practice. Model accuracy can be influenced by differences in IVUS machines, pullback speeds, and operator techniques, which are important real-world factors that may affect reproducibility and reliability across centres. A challenge for clinical adoption is that automated AI measurements can be difficult for clinicians to validate manually, potentially limiting trust and uptake unless clear visual overlays or explainable outputs are developed. Combining IVUS with other imaging modalities, such as OCT or near-infrared spectroscopy (NIRS), may further enhance the accuracy of AI-driven assessments and provide a more comprehensive evaluation of coronary lesions. While AI has improved the detection of overall plaque burden, accurately identifying vulnerable, high-risk plaques remains a more challenging and clinically critical task, as these are the lesions most likely to cause adverse events.

Post-procedure

Hyun-Seok Min et al. developed IVUS-based algorithms to predict post-procedural stent area, a critical factor in determining stent failure after drug-eluting stent placement. The models, trained on 28,952 IVUS frames from 515 patients, demonstrated a strong correlation between predicted and actual stent areas (r = 0.802, p < 0.001) and stent volumes (r = 0.958, p < 0.001). The algorithms achieved 94% accuracy in predicting stent under-expansion, supporting personalized pretreatment strategies to mitigate the 0.6% risk of late stent thrombosis within three years. This accuracy was achieved in internal validation only; external, multicenter testing is still needed to confirm generalizability and real-world performance and longer-term cardiac event risks [19]. Many studies in this area, including those by Zhu et al. and Bajaj et al., rely on small, single-center datasets, which limits the generalizability of their findings and highlights the need for larger, more diverse cohorts in future research.

Optical coherence tomography

OCT is an advent catheter-based imaging system allowing for analyses of the coronary luminal architecture and is complementary to angiography, which is used in calcium scoring of coronary vessels to quantify the degree of stenosis in the vasculature and determine the viability of them for stent placement. The applications of AI in OCT are severely limited in number; thus in this section we will focus on exploring the scope of applications of OCT in imaging using the models from a few existing studies that show the potential for further scope.

The first study, by Akiko Fujino et al., is a retrospective study using two sets of patients. The first set of 128 patients was used as a training set, and an external cohort of 133 patients was used as the determinant set. In the coronary calcification score, there were three parameters defined as maximum angle, maximum thickness, and length of the calcification, which were used to make a scoring system with a score range from 0 to 4, 0 being the least degree of stenosis and 4 being the most. Vasculature with scores 0-3 had adequate stent expansion upon stent placement, whereas arteries with a score of 4 had inadequate stent expansion. Models could be developed to directly assess the degree of stenosis, creating a percentage chance that a stent placement would be helpful in revascularization and assessing the risks in a subjective or objective grading system. The models can use various parameters such as the ones used to add to the objectivity of the judgmental analysis the model is theorized to do. The AI model can implement other factors such as FFR to boost statistical backing of AI grading of vasculature [53].

In the second study, Eisuke Usui et al. investigated whether features of unstable (vulnerable) plaques detected by OCT imaging in 340 patients (covering 382 lesions) were associated with physiological impairment, as measured by FFR and the index of microcirculatory resistance. The study aimed to determine if integrating plaque instability patterns from OCT could improve physiologic assessment of coronary lesions.. The development of AI models can implement the prevalence to aid in degree of physiological stenosis and add an extra criterion to the grading of vasculature and its subsequent use of its vulnerability and viability for interventional procedures for revascularization [54]. In this context, AI could integrate patterns of plaque instability detected by OCT into physiologic stenosis prediction models, enhancing the assessment of lesion vulnerability and functional significance. Other applications of OCT are within the same problems and variables that link to physiological stenosis due to various lesions that are judged using quantifiable parameters. Other studies’ authors compare the viability of OCT against IVUS and angiography, with most finding OCT to be superior [15] as OCT offers higher spatial resolution than IVUS and angiography, enabling more detailed plaque characterisation, precise measurement of fibrous cap thickness, and better visualisation of stent apposition and edge dissections.

With AI, it is clear now that the errors in human interpretation of complex imaging data can be reduced. For example, AI algorithms have better accuracy in identifying and measuring plaques such as fibrous caps or lipid-rich cores using OCT images that are necessary for assessing the severity of atherosclerosis [55]. Such a level of resolution helps also in intraoperative guidance, which can assist the surgeons to place grafts at ideal locations. The intraoperative navigation and guidance offered by AI allows surgeons to look at the patient’s anatomy in a real-time 3D format by using extremely advanced visualisation systems conjugated with AI technology. The use of AI extends to the postoperative phase of CABG patients’ management. In fact, patient outcomes can be enhanced with AI-driven models that detect and predict complications such as kidney injury or atrial fibrillation, permitting interventions ahead of time. Further, remote monitoring systems using AI to convey information instantaneously allow for the detection of adverse events, which can reduce hospital readmission [56]. However, widespread adoption is limited by the lack of standardised, expertly labeled OCT datasets for training, and by the technical challenge of integrating AI algorithms into real-time cath lab workflows. Emerging AI applications in OCT include automated prediction of restenosis risk, detection of calcified nodules, and advanced plaque composition analysis, which could further personalise interventional strategies, notable examples include the Ultreon platform (Abbott), which uses deep learning for real-time calcium quantification, and other convolutional neural network-based systems designed for automated plaque and stent analysis. Most AI-OCT applications remain in the proof-of-concept stage, with limited external validation across diverse patient populations and imaging systems. Additionally, the high computational demands of deep learning models and the need for seamless integration into existing OCT consoles present significant technical barriers to routine clinical use. It should be noted that OCT is not typically used intraoperatively during CABG procedures; its primary role remains in coronary interventions.

## Conclusions

This narrative review has gone through the various applications of artificial intelligence for various interventional cardiology procedures at multiple steps of patient management and identified credible and reliable applications. Most of the studies use a narrow patient criteria to lower failure rate (meaning that the program does not work) of the algorithm, this should be circumvented in upcoming studies by using more data, more variables and more inclusion criteria which will improve the accuracy of the algorithms itself and be used to develop improved ones that can be applicable into patient management. However, increasing the scope of inclusion criteria and expanding datasets introduces challenges such as greater heterogeneity and the potential for algorithmic bias, particularly if certain patient groups are underrepresented or data sources are inconsistent and to address these issues, future AI models should be developed and validated using multicenter, prospective trials with diverse patient populations, ensuring that algorithms are both accurate and generalisable across different clinical settings. AI should be empowering clinicians and not replacing them. AI has been shown to improve clinical maneuvering in real time and can only be further implemented by acting as a much more useful tool for surgeons in the future. 

Real-time guidance during interventional cardiology practice in the various procedures discussed there is a large scope of further research. While AI is a major asset, it will make sure clinicians never have to guess or more like make more of an educated guess and embody the “experienced guess” which clinicians accumulate over years of their practice. There should be studies for AI to cover more cases, normal and abnormal, to develop a sense of guesstimates to allow the program to not only use primary variables to predict outcomes but every aspect about the case to aid in procuring the equivalent to a clinician’s educated guess, while at the same time preserving the clinician’s command. Artificial intelligence is increasingly enabling more precise assessment of patient risk, dynamic guidance during complex procedures, and enhanced monitoring in the recovery phase-contributing to safer and more individualised care. Yet, widespread adoption is tempered by key hurdles, including the need for transparent and interpretable models, clearly defined legal responsibility in clinical settings, and evolving regulatory standards that govern the safe integration of AI into healthcare workflows. Although AI can improve clinical decision-making, AI has proven to comfortably predict postoperative complications accurately, making it reliable to clinicians, which should improve in the future. Despite these advances, important barriers remain, including the need for explainable AI models that clinicians can trust, unresolved medico-legal questions about responsibility for AI-driven decisions, and evolving regulatory standards that must be met before widespread clinical adoption can occur.
